# POGs2: A Web Portal to Facilitate Cross-Species Inferences About Protein Architecture and Function in Plants

**DOI:** 10.1371/journal.pone.0082569

**Published:** 2013-12-10

**Authors:** Michael Tomcal, Nicholas Stiffler, Alice Barkan

**Affiliations:** Institute of Molecular Biology, University of Oregon, Eugene, Oregon, United States of America; Lawrence Berkeley National Laboratory, United States of America

## Abstract

The Putative orthologous Groups 2 Database (POGs2) (http://pogs.uoregon.edu/) integrates information about the inferred proteomes of four plant species (*Arabidopsis thaliana*, *Zea mays*, *Orza sativa*, and *Populus trichocarpa*) in a display that facilitates comparisons among orthologs and extrapolation of annotations among species. A single-page view collates key functional data for members of each Putative Orthologous Group (POG): graphical representations of InterPro domains, predicted and established intracellular locations, and imported gene descriptions. The display incorporates POGs predicted by two different algorithms as well as gene trees, allowing users to evaluate the validity of POG memberships. The web interface provides ready access to sequences and alignments of POG members, as well as sequences, alignments, and domain architectures of closely-related paralogs. A simple and flexible search interface permits queries by BLAST and by any combination of gene identifier, keywords, domain names, InterPro identifiers, and intracellular location. The concurrent display of domain architectures for orthologous proteins highlights errors in gene models and false-negatives in domain predictions. The POGs2 layout is also useful for exploring candidate genes identified by transposon tagging, QTL mapping, map-based cloning, and proteomics, and for navigating between orthologous groups that belong to the same gene family.

## Introduction

The ability to acquire genome sequence data far outpaces the ability to infer gene functions. The vast majority of functional data comes from work with a handful of intensively studied model organisms, each of which is represented by a species-specific database that compiles functional data in the context of predicted and validated gene models. For genes that have not been studied directly, extrapolation of functional data from orthologous genes is a powerful method for inferring function, and comparisons of orthologous gene models can make errors in gene models apparent. The information for such comparisons can be extracted from existing species-specific and phylogenetic resources [1–6], but this requires time-consuming navigation between databases or among numerous pages within the same database. Thus, there is a need for web interfaces that bring together functional and structural annotations for orthologous genes in a manner that simplifies cross-species comparison and functional inference. 

In 2007, we developed the Putative Orthologous Groups (POGs) database to meet this need for plant genomes [7]. POGsDB was structured around the inferred proteomes of *Arabidopsis thaliana* (Arabidopsis) and *Oryza sativa* (rice), the two plant species whose complete genome sequences were available at that time. POGsDB collated data from multiple resources into a single-page format that allowed users to view salient functional information for orthologous proteins in rice and Arabidopsis at a glance. Here, we describe a major update, POGs2. POGs2 retains the user-friendly single-page view and flexible search strategies of the original POGsDB, but incorporates two additional plant species [*Zea mays* (maize) and *Populus trichocarpa* (poplar)], two independent sets of ortholog predictions, and numerous interface improvements as described below. 

## Methods, Results, and Discussion

### Overview

The POGs2 database is a MySQL database that organizes information about the inferred proteomes of Arabidopsis, rice, maize, and poplar in the context of Putative Orthologous Groups (POGs). Because large-scale orthology prediction is a developing art [8], we imported orthologous groups from two sources that employed different methodologies: orthologs imported from Gramene (http://www.gramene.org) [2] were calculated with the Ensembl pipeline [9,10], and orthologs imported from PLAZA (http://bioinformatics.psb.ugent.be/plaza/) [11] were calculated with OrthoMCL [12]. Gramene-calculated POGs are displayed by default, accompanied by a statement indicating whether the OrthoMCL prediction from PLAZA is in agreement; if not, the interface allows easy navigation between the alternative POG predictions. 

Other data in the POGs2 database was drawn from the following sources. Inferred proteomes with associated gene descriptions and InterPro domains [13] were imported from: *Arabidopsis thaliana* genome v10, ftp://ftp.arabidopsis.org/; *Oryza sativa* genome v7, ftp://ftp.plantbiology.msu.edu; *Zea mays* genome B73 Release 5b (http://ftp.maizesequence.org); *Populus trichocarpa* genome v8 (ftp://ftp.jgi-psf.org/pub/compgen/phytozome/v8.0/Ptrichocarpa/). Experimentally-validated intracellular locations of maize and Arabidopsis proteins were imported from the Plant Proteome Database (http://ppdb.tc.cornell.edu/‎) [14]. InterPro domains are displayed as graphics drawn from a custom graphics library written in PHP. The library takes the start and end locations of each annotated domain within a gene model, and renders an image scaled to the length of the protein. Each domain is illustrated as a colored ellipse, with the color alternating for each InterPro algorithm. Domain graphics are linked via an image map to the InterPro site for that particular domain. 

### User Interface: POG-view page

**Figure 1:**
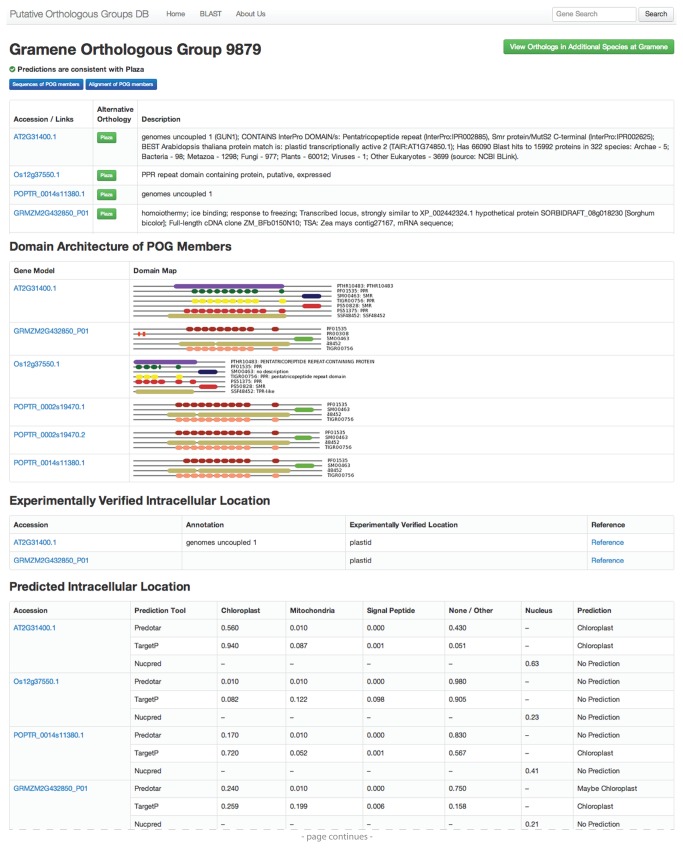
Screen capture showing upper portion of a representative POG page. Users can quickly assess that this POG prediction is likely to be valid because Gramene and PLAZA predict the same orthologous group (see top), and the members of the group form a clade in the tree (see asterisks in tree at bottom of Figure 2). The layout highlights the orthology of the uncharacterized maize and rice proteins to the characterized Arabidopsis protein GUN1, even though the gene descriptions fail to capture this relationship. The graphical illustration of domain architecture reveals what is likely to be an incorrect gene model in rice. The bottom portion of the same page is shown in Figure 2.

**Figure 2:**
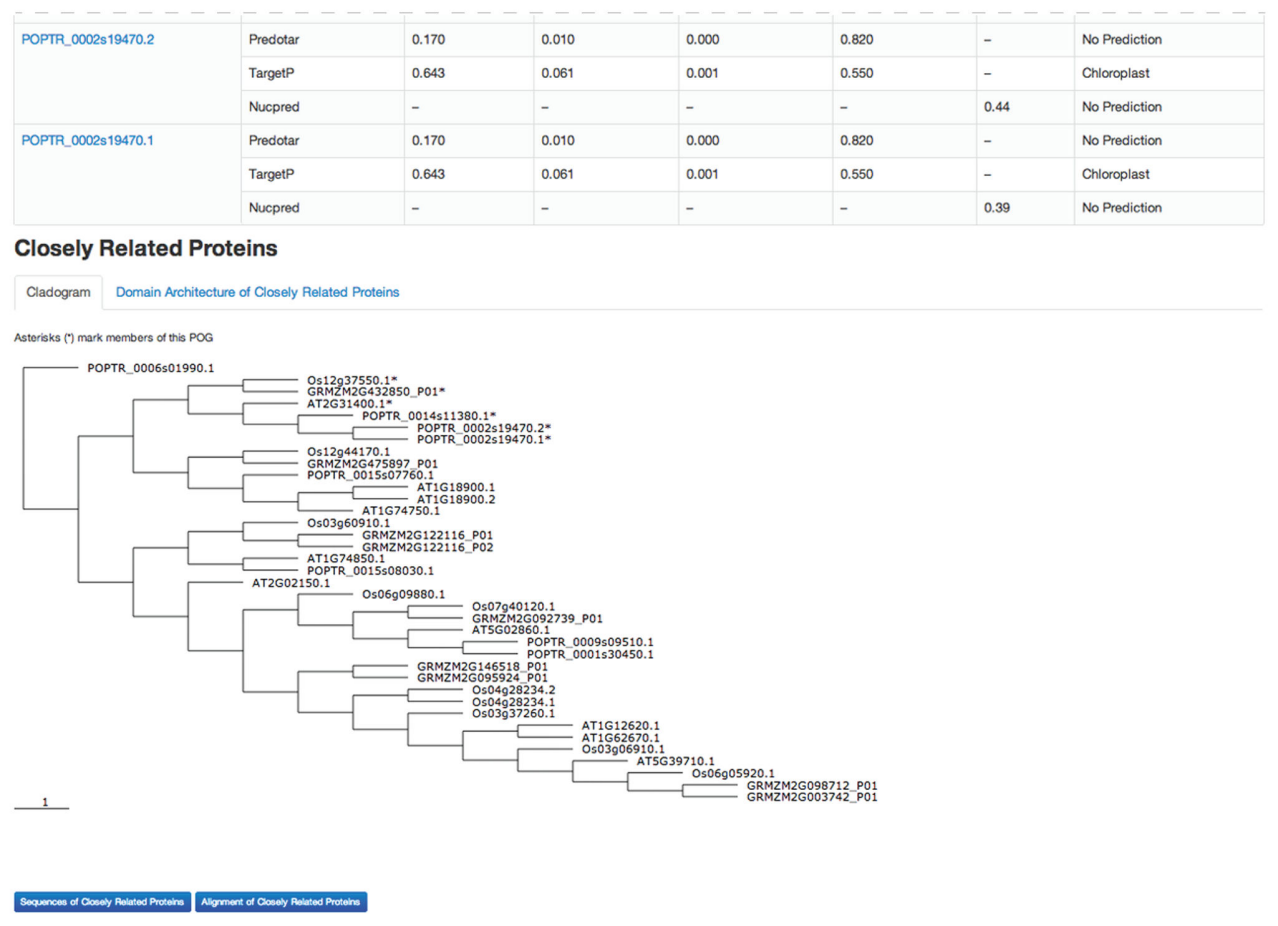
Screen capture showing bottom portion of a representative POG page. This is the bottom portion of the page shown in Figure 1.

By collaborating closely with experimentalists whose research requires frequent access to this type of data, we developed a user-friendly interface for searches, data visualization, and navigation to complementary web resources. A single-page format allows users to quickly assess key functional and phylogenetic features of POG members without the need to navigate to multiple pages (Figures 1 and 2). Each “POG-view” page includes the following information about each POG member (Figure 1): i) Gene identifiers and imported gene descriptions, with links to each gene in the corresponding species-specific database; ii) Graphical displays of domain architectures, with links to the corresponding InterPro domain descriptions; iii) Experimentally verified intracellular locations; iv) Subcellular targeting predictions for mitochondria, chloroplasts, and the secretory system calculated with TargetP v1.1 [15] and Predotar v1.03 [16], and for the nucleus calculated with NucPred [17]. This information is spatially organized according to data type rather than to gene, in order to facilitate comparisons among orthologs (Figure 1). 

**Figure 3:**
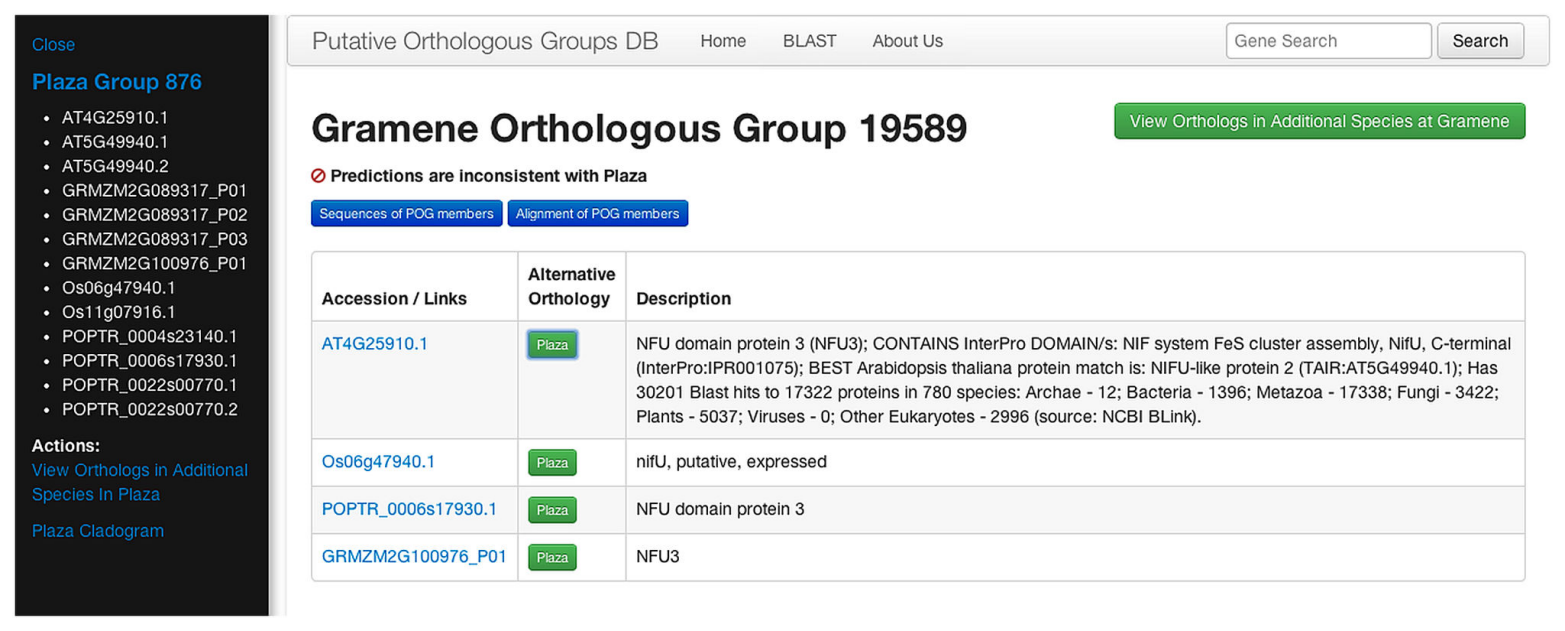
Example of sidebar showing alternative orthology predictions when OrthoMCL and Gramene predictions are inconsistent. The PLAZA Group identifier in the side bar is a link to the full POG-view page for that orthologous group.

Because the validity of the ortholog predictions is essential to support valid cross-species inference, two tools are provided to help users assess the quality of the predictions. First, a statement at the top of each POG-view page indicates whether the Gramene and PLAZA predictions are in agreement (Figure 1); if not, buttons next to each gene bring up a side-bar showing the corresponding orthologous group from PLAZA (Figure 3). Second, a tree is displayed at the bottom of each POG page with the POG members marked with asterisks (Figure 2); this allows users to quickly assess whether the POG members cluster as expected if they are orthologous. To generate these trees, proteins that are related to POG members were retrieved from an all-against-all BLAST search employing the first gene model for each gene (e.g. Os06g51110.1) as query against the inferred proteomes of the four species in the database. Matches with an E-value < 1e-10 were retained and the top four hits for each POG member (denoted “Closely Related Proteins”) were used to generate multiple sequence alignments with MUSCLE [18]. MUSCLE uses UPGMA to generate phenetic trees during its calculation of multiple sequence alignments. These trees were retrieved, simplified by restricting them to the first two gene models for each gene in the “closely-related” set, and displayed using jsPhyloSVG [19]. Each gene in the tree is represented by a link through which users may navigate to related POGs. 

**Figure 4:**
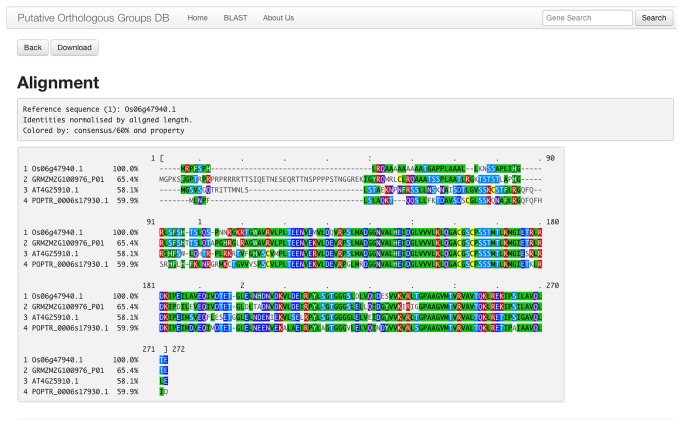
Screen capture of a multiple sequence alignment of POG members. Alignments are viewed by clicking a button at the top of the POG-view page (see Figure 1), and can be downloaded in several different formats. Amino acids are shaded according to their chemical properties to draw attention to potential functional features.

Buttons on each POG-view page allow users to view or download sequences and multiple sequence alignments of POG members (Figure 1, top), as well as sequences, multiple sequence alignments, and domain architectures of closely-related paralogs (Figure 2, bottom). Multiple sequence alignments were calculated with MUSCLE [18] and are displayed with MView [20] using a coloring scheme that reports the chemical properties of each amino acid (i.e. acidic, basic, polar, uncharged, etc) to highlight functional similarity (Figure 4). Gene identifiers for putative orthologs in species beyond the four represented in the POGs2 database can be accessed via links to the corresponding orthologous group in Gramene and PLAZA.

POGs2 employs a client-side JavaScript library AngularJS (http://angularjs.org) to retrieve JSON data files from our PHP API server, and render them into text and graphics By using this method, POGs2 relies largely on the web browser for rendering data rather than relying on the server alone. Thus, the load time is very quick and the server may handle numerous requests simultaneously without a significant decrease in speed.

### User Interface: Search Page

**Figure 5:**
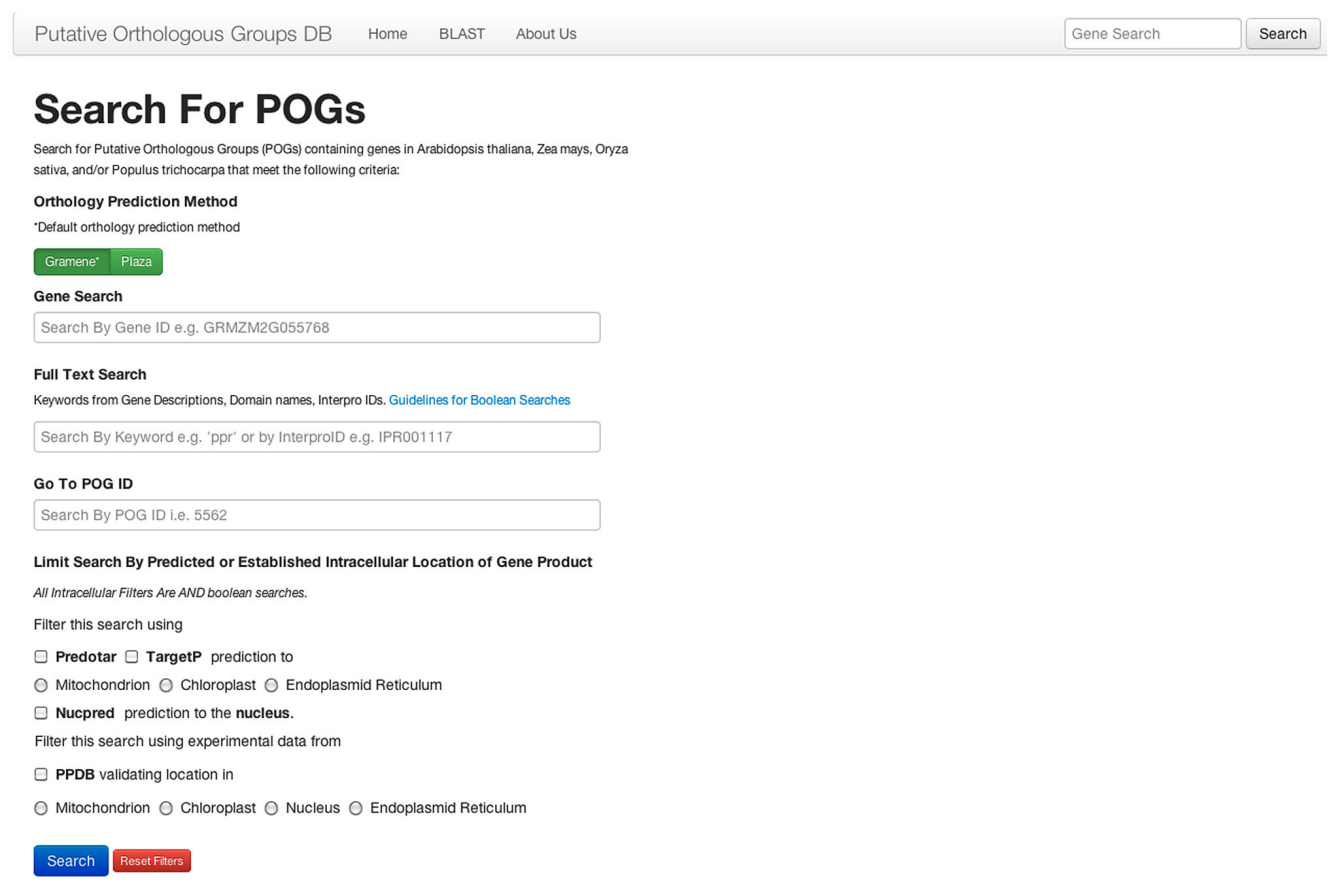
Screen capture of the POGs2 search page. Gene identifiers from any of the four species represented in the database may be used as a query under Gene Search. Searches for specified domain content, key word, and predicted or established intracellular location may be combined.

POGs2 offers flexible and intuitive options for queries based on gene identifier, keywords, domain names, InterPro domain identifiers, and predicted or established intracellular locations (Figure 5). The keyword search features Boolean operations, with “and” being the default operator when multiple search terms are used. Wildcard searches may be added to a full text query by adding an asterisk (e.g. ‘ribosom*’). A BLAST interface [21] provides access to POGs harboring proteins that are related to a query sequence. To achieve a rapid, responsive search interface we employed Sphinx (http://sphinxsearch.com/) for full text indexing of annotation data, and recent search results are cached in the key-value storage engine Redis (http://redis.io/). 

### POGs2’s Niche Among Genome Database Resources for Plants

POGs2 complements existing database resources for plant genomics by collating a subset of data types available elsewhere in a manner that simplifies protein-centric cross-species functional inferences. POGs2 is distinct in the types of data that are displayed concurrently for orthologous proteins (gene descriptions, domain architectures, intracellular locations, protein trees), in the ease of accessing protein sequences and multiple sequence alignments for orthologous proteins, and in the types of searches that are readily accomplished (e.g. searches for orthologous groups based on both conserved domains and intracellular location). Whereas other resources provide greater depth of information about particular species or greater breadth of phylogenetic data, POGs2 offers a rapid first stop for researchers wishing to glean the essence of what is known about the functions of a particular protein and its orthologs. For this reason, the display is limited to four model plant species, and priority was placed on devising a highly responsive interface that minimizes the need to navigate to multiple pages. Links are provided to species-specific and phylogenetic resources for those wishing to explore more deeply or broadly. 

POGs2 is particularly useful as the starting point for evaluating and prioritizing candidate genes that arise through genetic, coexpression, or proteomic analyses. QTL mapping, transposon-tagging, and map-based cloning approaches for identifying genetic variations that underlie phenotypes typically yield multiple candidates. Likewise, coexpression and coimmunoprecipitation data yield sets of genes that are candidates for harboring functions of interest. It is typically both time-consuming and costly to follow up all such candidates, so informed prioritization is a worthwhile first step. By bringing together gene descriptions, domain architectures, and intracellular location data for orthologous proteins in a single page view, and by providing ready link access to coexpression and publication data in Arabidopsis, users working in both “model” and “non-model” plant species can quickly distinguish the more promising from the less promising candidates. This aspect has been enormously useful in our own efforts to characterize the gene complement underlying chloroplast biogenesis via a large-scale forward genetic approach in maize [22]. In fact, this was our primary motivation for developing POGs2, as we found that existing online resources (i) required time-consuming multi-page navigation to access the core functional data that is collated in POGs2; and/or (ii) included a wealth of detail that hindered the extraction of the core functional data. 

Other benefits of the POGs2 layout include the ease of access to protein sequences and multiple sequence alignments, and graphics that display domain architectures in a manner that highlights likely errors in gene models and false-negatives in domain predictions (see Figure 1). In the future, we plan to incorporate additional proteome data, direct links to selected coexpression databases, button access to graphical representations of expression profile data, and refined POG predictions as methods for large-scale ortholog prediction continue to improve.
